# On Protein Loops, Prior Molecular States and Common Ancestors of Life

**DOI:** 10.1007/s00239-024-10167-y

**Published:** 2024-04-23

**Authors:** Kelsey Caetano-Anollés, M. Fayez Aziz, Fizza Mughal, Gustavo Caetano-Anollés

**Affiliations:** 1 Evolutionary Bioinformatics Laboratory, Department of Crop Sciences and Carl R. Woese Institute for Genomic Biology, University of Illinois at Urbana-Champaign, Urbana, IL 61801 USA; 2 Callout Biotech, Albuquerque, NM 87112 USA

**Keywords:** Folds, Tree of life, Molecular evolution, Origin of life, Phylogenomic analysis, Structural domains

## Abstract

The principle of continuity demands the existence of prior molecular states and common ancestors responsible for extant macromolecular structure. Here, we focus on the emergence and evolution of loop prototypes – the elemental architects of protein domain structure. Phylogenomic reconstruction spanning superkingdoms and viruses generated an evolutionary chronology of prototypes with six distinct evolutionary phases defining a most parsimonious evolutionary progression of cellular life. Each phase was marked by strategic prototype accumulation shaping the structures and functions of common ancestors. The last universal common ancestor (LUCA) of cells and viruses and the last universal cellular ancestor (LUCellA) defined stem lines that were structurally and functionally complex. The evolutionary saga highlighted transformative forces. LUCA lacked biosynthetic ribosomal machinery, while the pivotal LUCellA lacked essential DNA biosynthesis and modern transcription. Early proteins therefore relied on RNA for genetic information storage but appeared initially decoupled from it, hinting at transformative shifts of genetic processing. Urancestral loop types suggest advanced folding designs were present at an early evolutionary stage. An exploration of loop geometric properties revealed gradual replacement of prototypes with α-helix and β-strand bracing structures over time, paving the way for the dominance of other loop types. AlphFold2-generated atomic models of prototype accretion described patterns of fold emergence. Our findings favor a ‛processual’ model of evolving stem lines aligned with Woese’s vision of a communal world. This model prompts discussing the ‘problem of ancestors’ and the challenges that lie ahead for research in taxonomy, evolution and complexity.

## Introduction

Three primary starting points of reason support evolution, *continuity*, *singularity,* and *memory* (Caetano-Anollés et al. 2018; modified from Wiley 1975). These axioms are inductive statements of the highest level of universality and explanatory power. One of them, *continuity*, has its roots in the *lex continui* (law of continuity) of Leibniz (1687), which Linnaeus summarized with the famous motto *‘Natura non facit saltum’* (nature does nothing in jumps) (Linnaeus 1751). The idea of continuity, the existence of an unbroken chain of entities or events, which Leibniz carefully elaborated at mathematical and philosophical level, has early origins in Greek philosophers but gains grounds in mathematics with infinitesimals, their replacement by the ‘limit concept,’ and recently their reinstatement with the rigorous ‘smooth infinitesimal analysis,’ which makes all mathematical functions continuous (Bell 2022). One intuitive formulation of the law is Leibniz’s principle of spatiotemporal continuity, the application of *lex continui* to time and space: *“Any change from small to large, or *vice versa*, passes through something which is, in respect of degrees as well as of parts, in between”* (Leibniz 1923; VI. vi. 56). Here, Leibniz consistently links concepts of continuity to contiguity, density, and possibility, terms that he consistently uses in his elaborations (Jorgensen 2009). In his letter to Bayle he also considers ‘transitions’ of any kind as continuous, especially in geometry and natural processes (Leibniz 1687). Epistemologically, this makes application of the principle of spatiotemporal continuity appealing to the realms of molecular and evolutionary biology, especially because its formulation involves a succession of events, the continuity of existence, and gradual change in the presence of saltation processes, all central elements of the evolutionary progression.

One grand challenge is to understand how the complex biochemical makeup of cellular machinery materialized in the course of evolution (Caetano-Anollés et al. 2008; Caetano-Anollés 2021). Two broad sets of heteropolymeric macromolecules represent the central information carriers of the cell, proteins, and nucleic acids, with the ribosome and its associated translation machinery acting as communicators between the two (Caetano-Anollés 2023). In the past few decades, the interface of structural and genomic biology uncovered an unprecedented but finite diversity of structural forms associated with these informational molecules. Over 1300 known protein folds, hierarchically cataloged in databases such as the Structural Classification of Proteins (SCOP) (Murzin et al. 1995) and SCOPe (Fox et al. 2014), currently portray the diversity of *structural domains*, the structural, functional, and evolutionary units of the protein world. The number of newly discovered folds is quickly vanishing with database growth, suggesting that protein classification is essentially complete at fold level of structural abstraction. Similarly, the Rfam database currently collects the diversity of RNA into over 4000 families (many united in clans), each represented by multiple sequence alignments, consensus secondary structures and covariance models (Kalvari et al. 2020). Given that both proteins and nucleic acids are macromolecules with more than one type of elementary unit (20 + amino acids or 4 + nucleotides, respectively), macromolecular change responsible for all of these innovations must unfold in a continuum of discrete steps imposed by the addition, subtraction, and/or modification of monomeric units or collections of them but tailored by novel structures, interactions, recruitments, rearrangements, and folding schemes (Caetano-Anollés et al. 2022).

Here, we revisit some recent structural phylogenomic studies of the natural history of protein loop structures (Aziz et al. 2023; Mughal and Caetano-Anollés 2023). We explore the mode and tempo of evolution of these prior molecular states, their associated functions, and their rise in common ancestors of life. We first dissect evolutionary chronologies of loop prototypes defined by their structure and geometry in proteins. We then make explicit the loop repertoires of the last universal common ancestor (LUCA) and the last universal cellular ancestor (LUCellA) of life. Our main objective is to understand how these ‘elemental architects’ unfolded the structural diversity of the protein world and contributed to the evolving cellular makeup of urancestors. We conclude by discussing the ‘problem of ancestors’ from taxonomy, evolution, and complexity points of view. Developing philosophical stances can help us better understand how prior molecular states contributed to the emergence of the many levels of biological organization.

## Prior Molecular States

Two bracketing scenarios can be invoked to explain the amazing structural diversity of proteins and nucleic acids: (i) an explosive and all-encompassing origin of structural forms at the beginning of life, 3.8 billion years ago (Gya), followed by gradual loss and diversification of individual molecular structures (mostly at sequence level) or (ii) gradual appearance of structural forms by processes of complexification operating at both sequence and structure levels. While these scenarios appear to revive the controversial debate between punctuated equilibria and phyletic gradualism (Gould and Eldredge 1977), there is however significant evidence aligned with scenario (ii) in which molecular innovations appear piecemeal in evolution. To begin with, a number of comparative genomic and phylogenomic analyses, including the generation of evolutionary chronologies and time-dependent networks, support the gradual rise of fold structures and functions in the protein world (reviewed in Caetano-Anollés et al. 2021). In particular, the phylogenomic reconstruction of a molecular clock of folds calibrated with biomarkers and geomarkers was able to link the geological record and fold evolution, showing that the protein world evolved by gradual accretion of fold structures (Wang et al. 2011). As was previously anticipated (Dupont et al. 2010), a clock of this type placed the evolution of biochemistry within a framework of planetary history. When reconstructing ‘trees of proteomes’ (with taxa representing proteomes) and trees of domains (with taxa representing domains), many structural innovations were found increasingly restricted to smaller groups of organisms as folds appeared later in evolution. This retrodictive pattern was also observed when reconstructing vectors of molecular features of hypothetical ancestors in these trees using character state reconstruction (CSR) methods. Note that phylogenomic reconstruction of trees of proteomes and domains materialized from *synapomorphies*, similarities arising from common ancestry. Chronologies of folds directly derived from trees of domains showed that folds present in all organisms appeared earlier than those that were specific to superkingdoms (Wang et al. 2007). These patterns revealed instances of gain and loss of innovations across lineages (Nasir et al. 2014, 2017). To illustrate, folds linked to multicellularity appeared massively after the rise of eukaryotic-specific folds (Caetano-Anollés and Caetano-Anollés 2005) and then unfolded gradually (Caetano-Anollés et al. 2021). Similarly, folds of scaffolding proteins linked to cell–cell communication, such as the membrane-associated guanylate kinases (MAGUKs), assembled their multidomain makeup with the rise of Metazoa (Wang and Caetano-Anollés 2009). Conversely, phylogenomic reconstructions of trees of proteomes from structural domains (Wang et al. 2007) and domain combinations in multidomain proteins (Wang and Caetano-Anollés 2006) showed fold distributions were increasingly scarce and limited to smaller groups of diversifying organisms toward the crown of the phylogenetic trees. A recent study reaches a similar conclusion. Tracing folds along the lineages of a timetree of cellular life reconstructed from sequence data, in which times of divergence were assigned to branch splits, revealed a substantial number of folds repartitioning in evolving lineages (Romei et al. 2022). The exercise showed eukaryotic folds specific to functions of cell motility appearing with Opisthokonta, functions of cell adhesion suggestive of early multicellularity with Filastera, signaling and apoptosis with Metazoa, adaptive immunity with Gnathostomata, and neurohormones and hemocyanins with Ecdysone (Romei et al. 2023). Fold synapomorphies associated with the roots (ancestors) of these clades manifested bursts of fold innovation. The coexistence of gradual change with bursts is an expected evolutionary outcome, as we have shown this coexistence in the combination of domains in proteins (Wang and Caetano-Anollés 2009) and the rise of metabolic networks (reviewed in Caetano-Anollés and Caetano-Anollés 2024). When modeling evolution of protein structure, simulations suggest evolutionary exploration of protein structure space occurs through coarse-grained discoveries that undergo fine-grained elaborations (Tal et al. 2016). These facts do not detract from compliance with a principle of spatiotemporal continuity. Bursts and gradual change represent two complementary but relative manifestations of the changing evolutionary landscape.

The principle of continuity also drives the emergence of molecular innovations. Macromolecules are known to grow over evolutionary time by accretion of component parts (Caetano-Anollés et al. 2022). One particularly illustrative example relates to the ribosome. While the structures of the ribosomes of archaeal and bacterial microbes do not depart much from the universal core (Melnikov et al 2012), the more recently solved ribosomal structures of *Tetrahymena thermophila* (protists) (Klinge et al. 2011), *Saccharomyces cerevisiae* (fungi) (Jenner et al. 2012), *Triticum aestivum* (plants) (Armache et al. 2010), *Drosophila melanogaster* (insects), and *Homo sapiens* (mammals) (Anger et al. 2013), in that order, are increasingly larger, with the large ribosomal subunit contributing the most to accretional growth. Phylogenomic analysis has shown that helical structures accrete in tRNA, 5S rRNA, RNase P RNA, SINE RNA, and rRNA as molecular structures unfold in evolution (reviewed in Caetano-Anollés and Caetano-Anollés 2015). The first studies of accretion made use of CSR along branches of a tree of life generated from rRNA (Caetano-Anollés 2002a) or direct reconstruction of trees of rRNA substructures describing ribosomal growth (Caetano-Anollés 2002b). These studies were later advanced to include evolution of ribosomal proteins, providing a very detailed phylogenomic-based model of ribosomal evolution and showing proteins and RNA were co-evolving in the ribosomal ensemble (Harish and Caetano-Anollés 2012). The model was contrasted against non-retrodictive algorithms of ribosomal dismantling driven by A-minor interactions (Bokov and Steinberg 2009) and of outward (onion) growth (Petrov et al. 2015) and against in silico-designed RNA ‘ring’ constructs mimicking ancestral molecules (Demongeot and Seligmann 2020a, b), showing approaches can be made to converge into congruency (Caetano-Anollés and Caetano-Anollés 2015). Remarkably, a principle of diminishing returns in RNA accretion history followed a tendency of sub-structural parts to decrease their size when molecules enlarge (Sun and Caetano-Anollés 2021). This tendency was best described by the Menzerath-Altmann’s law of language in full generality and without interference from the details of molecular growth.

An accretion process implies the existence of *prior molecular states*, intermediate forms (building blocks) that mediate the growth of the evolving macromolecules. Generally these prior states must behave as agential *dynamic systems* (Caetano-Anollés 2023). They must act as sets of autonomous, integrated, coordinated, and stable interacting parts (structural states) that can be characterized by their dynamic behavior and individuated by cohesion. Prior molecular states must also be evolutionary conserved, i.e., their dynamic stability must be such that has become persistent. Conservation allows to identify their prior existence as remnants of their presence in extant macromolecules. Some prior states may also behave as *modules*. Modules are groups of structures and processes linked more extensively to each other than to the rest of the system (Simon 1962). Their ability to perform tasks helps the biological system adapt and innovate by recruiting them multiple times in different contexts. Modularity enhances persistence by module reuse through recruitment, improving the flexibility and evolvability (Caetano-Anollés et al. 2022). Its absence makes structure and function monolithic (Caetano-Anollés 2021).

**Fig. 1:**
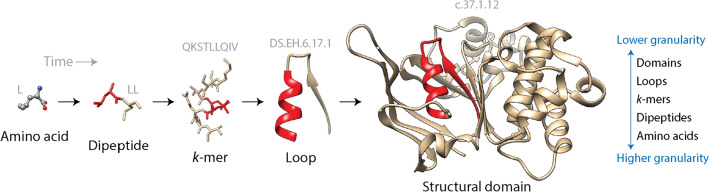
A chronology of prior molecular states of protein structural domains is supported by complementary scalar (compositional) and subsumption (historical) hierarchies that describe levels of structural organization. The figure illustrates the successive nesting of amino acid residues, dipeptides, *k*-mer sequences, loop structures, and domains using the ABC transporter ATPase domain-like (c.37.1.12) domain of the cobalt transport system of *Thermotoga maritima* (PDB entry 2YZ2, chain A) as an example. Red substructures make explicit the scalar hierarchy, which nests differently sized dynamical entities into each other by mereologically defining parts and wholes as [[part] whole] statements and invoking ‘is-a-part-of’ relationships between them. Nesting relationships are compositional in their logic. In the example, they embed smaller parts into larger wholes outwardly toward higher levels of the hierarchy and without an integrative or historical rationale. Adding a temporal framework (arrows) produces a subsumption hierarchy that defines a taxonomy of the dynamical system with [general[specific]] statements and a “is-a-kind-of” ordering principle between levels of organization. This hierarchy demands establishing history through “intermediate forms” or “prior states” of a refining, developing or evolving system. Here, amino acids ⊂ dipeptides ⊂ *k*-mers ⊂ loops ⊂ domains, where ⊂ indicates “is a subset of.” The two complementary logical forms of hierarchical structure capture more broadly Simon’s view of hierarchies being described by “states” (the world as sensed), which defines observables, and hierarchies described by “processes” (the world as acted upon), which prompt the system to act “purposefully upon its environment” (Simon 1962). The “states” and “process” views stress the unification or the diversification of the system, respectively

Figure 1 illustrates prior molecular states that mediate the evolution of proteins. These prior molecular states define levels of molecular organization that fulfill the tenets of ordering relationships typical of hierarchical organization (Fig. 1). Here, hierarchical structure is established by ranking parts (temporal parts sensu Caetano-Anollés et al. 2022) of a system against each other and establishing authorities and boundary conditions (Salthe 1985, 2012). The resulting nesting relationships give rise to scalar (compositional) and subsumption (historical) hierarchies. The folds of the structural domain units are delimited by *loops*, a diverse group of building blocks made of helix, strand, and return regions of the polypeptide chain that are evolutionarily conserved and important for protein function and flexibility (Papaleo et al. 2016). These loop structures embed in them more primordial *k*-mer motifs, which have been shown to distinguish genomes (Pe’er et al. 2004) and carry signatures of history (Choi and Kim 2020). Finally, *k*-mers also embed in them dipeptides in the form of concatenated *2*-mer amino acid sequences. These dipeptides make up a set of 400 + primordial building blocks defining the contextual framework of the peptide bond, the chemistry that makes a protein possible. Dipeptide compositions act as powerful proteomic signatures of thermophilic, halophilic, and pH adaptations to extreme environments (Amangeldina et al. 2024) They also reveal hidden links between protein structure and the genetic code that are mediated by protein flexibility (Caetano-Anollés et al. 2013). In fact, it has been well known for some time that amino acid compositions determine the fold structure of proteins (Nakashima et al. 1986). Note that these prior molecular states are for the most part embedded in the makeup of a protein. However, they also exist as free-standing units in the extant world. For example, while dipeptides can be identified as *2*-mer units in proteins they are also free-standing molecules produced by the non-ribosomal peptide synthetase (NRPS) machinery of the cell, many of which are indexed by the Norine database (Flissi et al. 2020). These NRPS-synthesized peptides are 2–26 monomers in length and are made from more than 500 different building blocks.

## Protein Loops: Stepping Stones to Protein Structure

Proteins fold cooperatively into many layers of molecular organization through asynchronous formation of secondary structures, co-translational stabilization of newly formed structures along the ribosomal exit tunnel, and long-distance interactions that guide the free-energy landscape toward the native state (Caetano-Anollés et al. 2021). Folds embed ‘closed’ loop structures, returns of the protein backbone that are much smaller than the typical ~ 100 amino acid residues of a typical domain, some of which represent well-known structural motifs, including α-hairpins, β-hairpins, and β-turns widely present in proteins (Levitt and Chothia 1976). A number of ‘supersecondary’ elements were first identified with structural alignment approaches, including antiparallel ββ, αββ, ββα, and αα-turn motifs (Presnell et al. 1992; Holm and Sander 1993; Boutonnet et al. 1998; Wintjens et al. 1996). This prompted a more general classification scheme of supersecondary motifs, which defined these so-called *Smotifs* as two regular secondary structure elements connected by a loop region in geometrically-specific orientations (Oliva et al. 1997; Fernandez-Fuentes et al. 2006). These types of supersecondary elements behave as autonomous structural, functional, and evolutionary units. There is significant evidence supporting these roles (Romero Romero et al. 2016; Berezovsky et al. 2017; Heizinger and Merkl 2021). Hidden Markov models (HMMs) and sequence profiles (patterns of amino acid conservation given a position-specific scoring matrices) derived from multiple sequence alignments (MSA) have identified evolutionarily conserved loop motifs that either stand alone or combine with others in proteins. Their conservation suggest they likely played a crucial role in the evolutionary development of molecular functions. Non-combinable loops are highly repeated structures 9–39 residue long that appear in popular folds and are claimed to represent remnants of ancient peptide building blocks that existed in a primordial RNA-peptide world (Alva et al. 2015). These loop motifs were identified by searching for local sequence and structural similarities in protein domains with the HHsearch and TM-align algorithms, respectively. Loops were enriched in catalytic dinucleotide, nucleic acid, and metal ion/iron sulfur cluster-binding functions. In contrast, combinable ‘closed’ loop motifs, typically 25–30 residue long (Berezovsky et al. 2000; Berezovsky and Trifonov 2001), formed active sites biding cofactors, and determining molecular functions (Goncearenco and Berezovsky 2015). These so-called elementary functional loops (EFLs) were identified by iterative derivation of profiles from protein coding sequences in complete proteomes with methods that resemble the PSI-BLAST procedure (Goncearenco and Berezovsky 2010). EFLs identified by these sequence profiles in archaeal proteins unified ABC transporters, methylases and methyltransferases, aminoacyl-tRNA synthetases and helicases, metal-binding proteins, transcriptional regulators, and cell surface proteins (Goncearenco and Berezovsky 2012). They appear to behave as ancient structural scaffolds that emerged in protein evolution from shorter protein modules. Indeed, an evolutionary chronology of EFLs and domains unfolding in bipartite networks showed that EFLs had their origins in nucleotide-binding P-loop motifs of the multifunctional α-β-α-layered design typical of ABC transporters holding the ‘P-loop containing nucleoside triphosphate hydrolase’ fold (Aziz et al. 2016). Most remarkably, the evolving networks also showed EFL recruitment events occurring throughout the EFL and domain timeline, suggesting the origin of novel EFLs and domains and their corresponding functions is an ongoing process. Finally, a third type of supersecondary motif include the so-called ‘theme’ (Nepomnyachiy et al. 2014, 2017). These conserved structural elements are 35 to 200 residue long. They are widely reused, contiguous, and non-overlapping. Networks have been constructed that link domains with themes showing how these motifs are being reused in proteins and revealing a large network component associated with domains holding alternating α and β elements of structure (Nepomnyachiy et al. 2014).

While supersecondary motifs, non-combinable loops, EFLs, and themes are considered conserved and likely ancient evolutionary building blocks of proteins, their identification exploits sequence and/or structure similarities that do not necessarily carry an evolutionary relationship. They do however show motifs recur across proteins, in some cases driven by biological function. This suggests the existence of a complex interplay between divergent and convergent evolutionary processes leading to recruitments, rearrangements, duplications, and divergences. Note that the use of machine learning allows to accurately identify sub-domain-sized fragments and their clustering into groups, which are often associated with specific functions.

## Retrodicting Protein Loop History

While prediction forecasts the future based on information available in the past, retrodiction explains past events based on information in the present. These endeavors involve either forward-time and reverse-time representations, which bare on the amount of information (excess entropy) that is stored in the present (Ellison et al. 2009). In other words, the present often holds enough information to predict and retrodict, a fact that is well known to evolutionary biologists that seek to advance systems and synthetic biology applications. Traveling back in time (metaphorically) can be accomplished experimentally and/or computationally, depending on time frames. Experimental retrodiction involves for example the ‘resurrection’ of hypothetical ancestors that were computationally defined by CSR methods (Zaucha and Heddle 2017), reviving viruses and microbes from ancient permafrost (Alempic et al. 2023) or preserving them in frozen laboratory conditions for ongoing or future research (McDonald 2019), extracting ancient DNA from amber, ice cores, or other materials, for example, to reconstruct paleogenomes (Shapiro and Hofreiter 2012), or tracking the genomic makeup of viruses in real time along a pandemic (e.g., Talenti et al. 2022; Tomaszewski et al. 2023). Computational retrodiction entails building phylogenetic trees with or without reticulations (phylogenies) from data and models of evolutionary change, rooting the trees and then, tracking change along their branches (Caetano-Anollés et al. 2018). These phylogenies explicitly describe the history of observable biological features of interest (characters) in data that is phylogenetically informative, i.e., homologies that comply with the *memory* axiom. The most powerful tree reconstruction method, the ‘search’ strategy, involves optimizing the fit of data along the branches of all trees that are possible according to some optimality criterion and a model of character state change and then rooting the optimal trees to establish the direction of evolutionary change. The most powerful and generic rooting approach is the Lundberg optimization, which places the root at the most parsimonious location (Lundberg 1972). The method invokes Weston’s generality criterion, which optimizes ancestral-derived homology relationships in nested patterns along branches of the trees (the ‘standard’ implementation) or makes use of a maximum or minimum state ancestor according to evolutionary considerations (the ‘ancestor’ implementation) (Caetano-Anollés et al. 2019). Note that the generality criterion is a direct rooting method that avoids resorting to an outgroup or a molecular clock model and invokes a minimum number of assumptions (Caetano-Anollés et al. 2018).

**Fig. 2:**
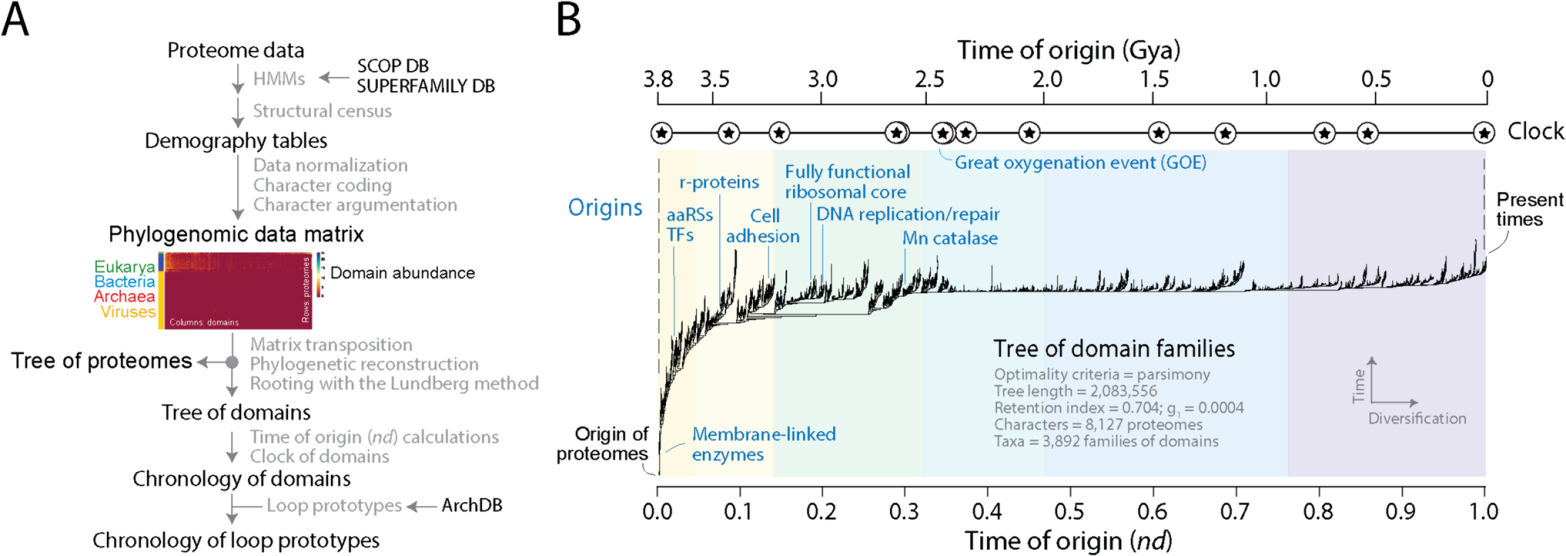
Reconstructing the evolutionary history of structural domains in the proteomes of cells and viruses. (**A**) Workflow showing the general phylogenomic reconstruction strategy. A census of SCOP domains driven by hidden Markov models (HMMs) of structural recognition (Gough et al. 2001) in the proteomes of thousands of completely sequenced genomes is used to build demography tables following published methods (Nasir and Caetano-Anollés 2015). In the present study, domain structures are defined at fold family level of structural abstraction in SCOP. Census data are then normalized and coded into data matrices of phylogenetic characters, here illustrated with an evolutionary heat map of the data used in panel B, with rows describing proteomes and columns describing domain families. Matrix elements (*g*) representing genomic abundances (with levels described with a scale) are converted into multi-state phylogenetic characters with character state changes following linearly ordered and undirected transformations (Caetano-Anollés et al. 2019). Trees of domains and trees of proteomes (representing Trees of Life) are generated from the matrices by transposition. These trees have domains or proteins as their leaves, respectively. Trees of proteomes are not used in the present study but organismal groupings are largely congruent with traditional classification. (**B**) A most parsimonious tree of domains describing the evolutionary history of 3892 domain families in 8127 proteomes sampling viruses and organisms from all major cellular taxonomical groups of the RefSeq database (O’Leary et al. 2016). The phylogenomic tree was built using PAUP* (Swofford 2023) with good performance (Kolaczkowski and Thornton 2004). A chronology was generated that ordered families according to their time of origin in a relative scale of ‘node distance’ (*nd*) or in billions of years ago (Gya) using a molecular clock of folds delimited by biomarkers and geomarkers (stars). Some domain families are indexed to show the rise of aminoacyl-tRNA synthetases (aaRSs), translation factors (TFs), ribosomal proteins (r-proteins), and other proteins of functional importance. Tree and chronology were generated by Mughal et al. (2020)

Macromolecular structure is evolutionarily conserved and can be effectively used to generate rooted phylogenomic trees describing deep protein evolution (Caetano-Anollés and Nasir 2012). Since the cornerstone of protein hierarchical classification is homology of structural domains, the existence of shared-and-derived features in the sequence, structure, and function of these ‘modules’ represents an entry point strategy to uncovering the history of other prior forms, including that of protein loops. Rooted trees of domains are highly unbalanced. This permits the calculation of a time of origin for each structural domain, either as a relative age measured with a node distance (*nd*) from the root to the leaves of the tree (Caetano-Anollés 2005) or in billions of years (Gy) according to a molecular clock of fold structures (Wang et al. 2011). Times of origin are then used to build evolutionary chronologies and uncover patterns of macromolecular accretion and diversification of domain history. Note that (1) branches (or internal nodes) of trees of domains represent diversification events that occur as structural innovations in domain structure spread in proteomes; (2) leaves of the trees (taxa) are structural elements of proteins, which in contrast with the typical trees of systematic biology, may not be diagnostic of organisms; (3) the criterion of primary homology that establishes correspondences arising from common ancestry rests on the feature of structure being studied (domains) and their genomic abundance levels, i.e., ordered multistate characters establishing serial homologies that are testable with Weston’s generality criterion of ‘nesting’; and (4) trees of domains represent by themselves models of structural evolution describing how the protein world adds new or previously discovered domain structures to enhance the repertoires of evolving proteomes. The generation of chronologies from trees of domains has been recently reviewed (Caetano-Anollés et al. 2021). Figure 2 shows a workflow of the general experimental strategy and a phylogenomic tree of fold families describing the evolution of SCOP domains structures. The tree of domains, which was reconstructed from a census of domain families in 8127 proteomes belonging to cellular organisms and viruses (Mughal et al. 2020) was here used as reference to establish a chronology of loop structures.

**Fig. 3:**
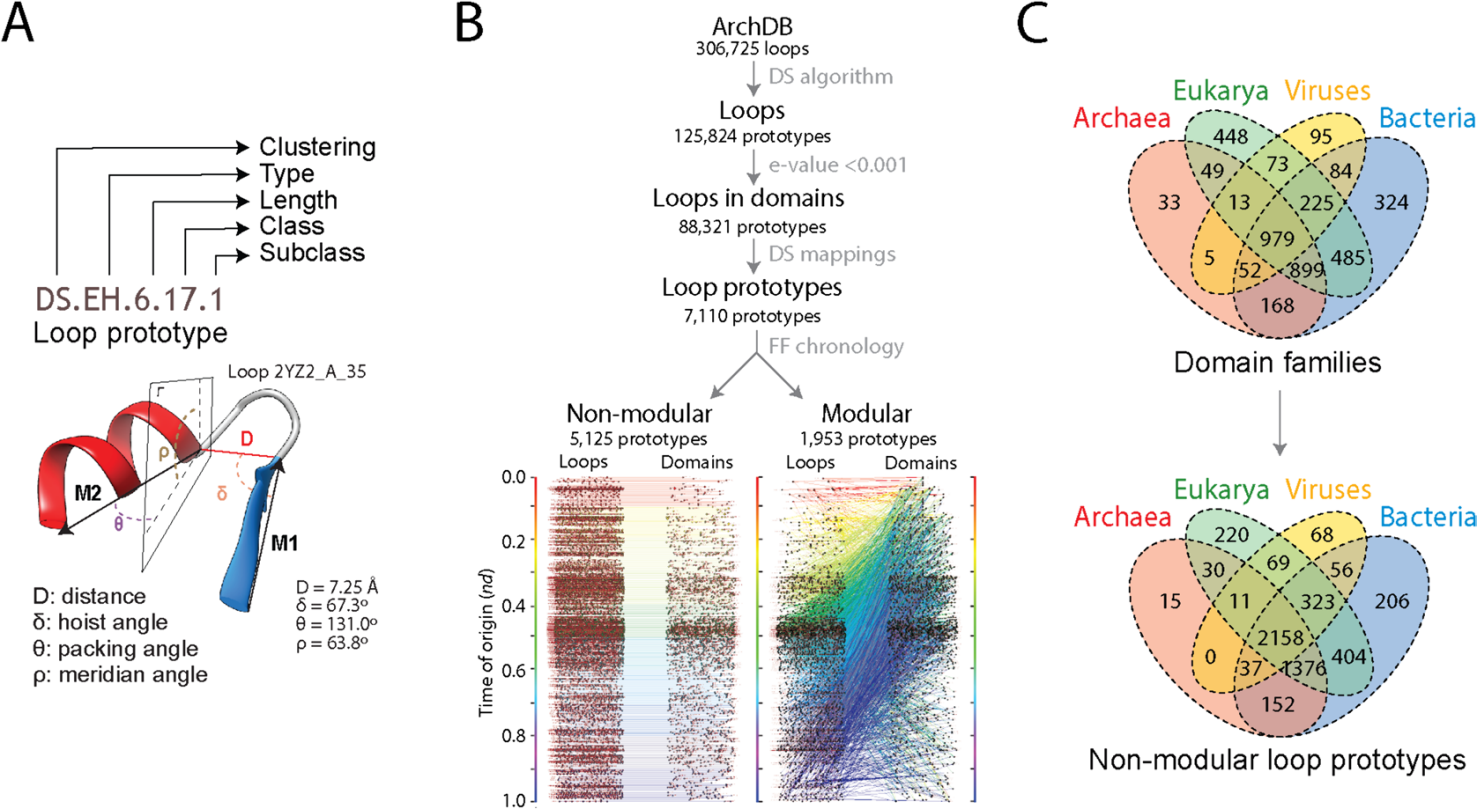
Exploring loop prototypes as prior molecular states. (**A**) The ArchDB defines a loop prototype by the clustering method used to unify loop structures into prototypes (DS or MCL), the bracing secondary structures of the loop (type), the number of residues forming the aperiodic structure (length), its conformation given as a Ramachandran consensus of φ and ψ backbone dihedral angles of the participating residues (class), and the geometry of the loop (subclass). We illustrate the 4-level tree-like classification system with the atomic model of loop 2YZ2_A_35 described in Fig. 1, which is part of prototype DS.HE.6.17.1. The geometric properties of the loop are measured using four internal coordinates (D, δ, θ, ρ) extracted from the orientation of principal vectors (M1 and M2) of the bracing secondary structures: the Euclidean distance in Ångstroms between the boundaries of the aperiodic structure (D), the angle between M1 and D (δ), the angle between M1 and M2 (θ), and the angle between M2 and the plane Γ defined by the vector M1 and the normal to the plane formed by M1 (ρ). (**B**) Mapping loop structures and prototypes to domain families allowed to place them along a chronology of domains. Prototypes that were *non-modular* or *modular* were mapped to domain families and displayed along separate chronologies as an evolving bipartite network of prototypes and domains. In this process, each prototype was assigned a time of origin (*nd*). Note the dense recruitment of M prototypes occurring in the middle of the timeline. Data from Aziz et al. (2023). (**C**) Four-set Venn diagrams describing the distribution of domain families and non-modular loop prototypes among superkingdoms of life (Archaea, Bacteria, and Eukarya) and viruses. Data from Mughal and Caetano-Anollés (2023)

We mapped loops sourced from ArchDB (Bonet et al. 2014a), a database that classifies loops based on geometry and conformation, to structural domains with the objective of tracing their history (Aziz et al. 2023; Mughal and Caetano-Anollés 2023). ArchDB loops are supersecondary structural motifs defined by two sequential periodic secondary structures connected by an aperiodic region. They represent basic units of classification extracted from known protein structural entries of the Protein Data Bank (PDB). ArchDB classifies loops based on the bracing secondary structures of the loop, the length of the aperiodic region, its conformation according to the ϕ and ψ backbone dihedral angles of its residues, and the geometry of the loop (i.e., the entire supersecondary structural motif) according to four internal coordinates (D, δ, θ, and ρ) (Fig. 3A). The tree-like classification hierarchy of ArchDB uses two clustering algorithms to automatically unify the loop structures into *loop prototypes* in the form of generalized representations of clustered loops defined at 4 classification levels (type, length, class, and subclass). The stringent Density Search (DS) algorithm detects regions of feature space with a high density of loops around a centroid defined by loop length, conformation, and geometry. The graph-based Markov clustering (MCL) algorithm simulates information flow along a network of loops connected by loop similarities. In our analysis we chose the more stringent DS clustering method because it limits the length of loops and enlarges the coverage of clustered groups.

Since each loop structure is annotated with a prototype, we filtered DS-defined prototypes (holding 125,824 loops) using mappings of prototypes to domain families at e-value < 0.001 (Fig. 3B). Note that the library of loop prototypes is essentially complete (Fernandez-Fuentes et al. 2010; Bonet et al. 2014b). All possible geometries have been sampled (Fernandez-Fuentes et al. 2010). In fact, saturation remains unchanged since year 2000 despite substantial growth of the PDB database. Similarly, the library of solved single domain protein structures is also complete (Skolnick et al. 2012). These finite systems make historical statements inferred from the mapping of loop prototypes to structural domains powerful because of their high level of universality. When mapping loop prototypes to domains along the chronology of domain families (Aziz et al. 2023), two types of prototypes were evident, *modular* and *non-modular*, with the number of non-modular prototypes exceeding 2.6 times that of modular prototypes (Fig. 3B). While modular prototypes mapped too many domain families along the timeline, non-modular prototypes mapped exclusively to domain families of a same time of origin. These mappings are remarkable but not surprising. A small set of popular prototypes in combination with many more rare ones were previously found capable of describing all folds that were known, including novel folds that were gradually added to the databases (Fernandez-Fuentes et al. 2010).

When prototypes were assigned times of origin and were displayed along separate chronologies as evolving bipartite networks (Aziz et al. 2023), the smaller set of modular prototypes revealed dense patterns of recruitment throughout the timeline, especially starting half way along the evolutionary progression (Fig. 3B). This shows the central evolutionary role that modular prototypes play in fold evolution. Visualizing the evolving network of modular prototypes as a ‘waterfall’ uncovered two primordial waves of functional innovation and recruitment involving founder ‘P-loop’ and ‘winged helix’ loop structures (Aziz et al. 2023). The P-loop prototype is embedded in the Rossmanoid α-β-α layered structure of the ‘P-loop containing nucleoside triphosphate hydrolase’ fold (SCOP *concise classification string*: c.37), which is the most ancient and popular in the evolutionary chronology of domains (Caetano-Anollés et al. 2021). It enables the crucial nucleotide triphosphate-binding functions of the folded structure. The helix-turn-helix (HTH) prototype typical of the ‘winged helix’ motif is embedded in the bundle structure of the ‘DNA/RNA-binding 3-helical bundle’ fold (a.4). The HTH prototype interacts with nucleic acids and proteins, playing central roles in transcription, clamping capacity to RNA polymerases, and domain–domain recognition in ubiquitin-ligase and condensing complexes. The two recruitment waves took advantage of cysteine-rich loop prototypes and strong recruitment pathways of three domain superfamilies necessary to develop translation, the ‘NAD(P)-binding Rossmann-fold domain’ (c.2.1), the ‘S-adenosyl-L-methionine-dependent methyltransferase domain’ (c.66.1), and the ‘OB-fold of the nucleic acid-binding protein domain’ (b.40.4). Note that the existence of these waves found corroboration at different levels of protein organization in the two oldest metabolic wheels of the first phylogenomic-driven study of metabolic evolution (Caetano-Anolles et al. 2007), chronologies of EFLs (Aziz et al. 2016), and chronologies of structural domain organization (Aziz and Caetano-Anollés 2021). It is remarkable that these recruitment waves involved the same primordial α-β-α-layered sandwich, β-barrels, and helical bundle structures identified as part of the first 54 domain families that appeared in evolution (Caetano-Anollés et al. 2012). Retrieval of congruent evolutionary history with different datasets provides strong support to our statements of metabolic evolution.

## Tracing the Origin and Evolution of Loop Prototypes Along a Chronology

To avoid untangling the evolutionary effects of recruitment, our analysis focused on the more numerous non-modular loop prototypes, which map one to one to domain families of a same age (Mughal and Caetano-Anollés 2023). As a first step, we determined how widely distributed were non-modular prototypes among the 8127 proteomes belonging to superkingdoms Archaea, Bacteria and Eukarya, and the Virus supergroup. These proteomes were used to generate the tree of domains of Fig. 2B. Figure 3C shows four-set Venn diagrams describing domain family and loop prototype distributions. While Venn groups somehow preserved the same distribution patterns in prototypes and families, including the high representation of prototypes and families common to all supergroups (the ABEV Venn group) and all superkingdoms (the ABE group), there were patterns that were notably distinct. While ABEV and ABE families were somehow comparable, ABEV prototypes were almost double those of the ABE group. Similarly, superkingdom-specific prototypes were significantly underrepresented when compared to families belonging to the A, B, and E groups. These comparative genomic findings already suggest enrichment of these widely shared prototypes may stem from them being more prevalent prior to the diversification of cellular organisms and viruses.

**Fig. 4:**
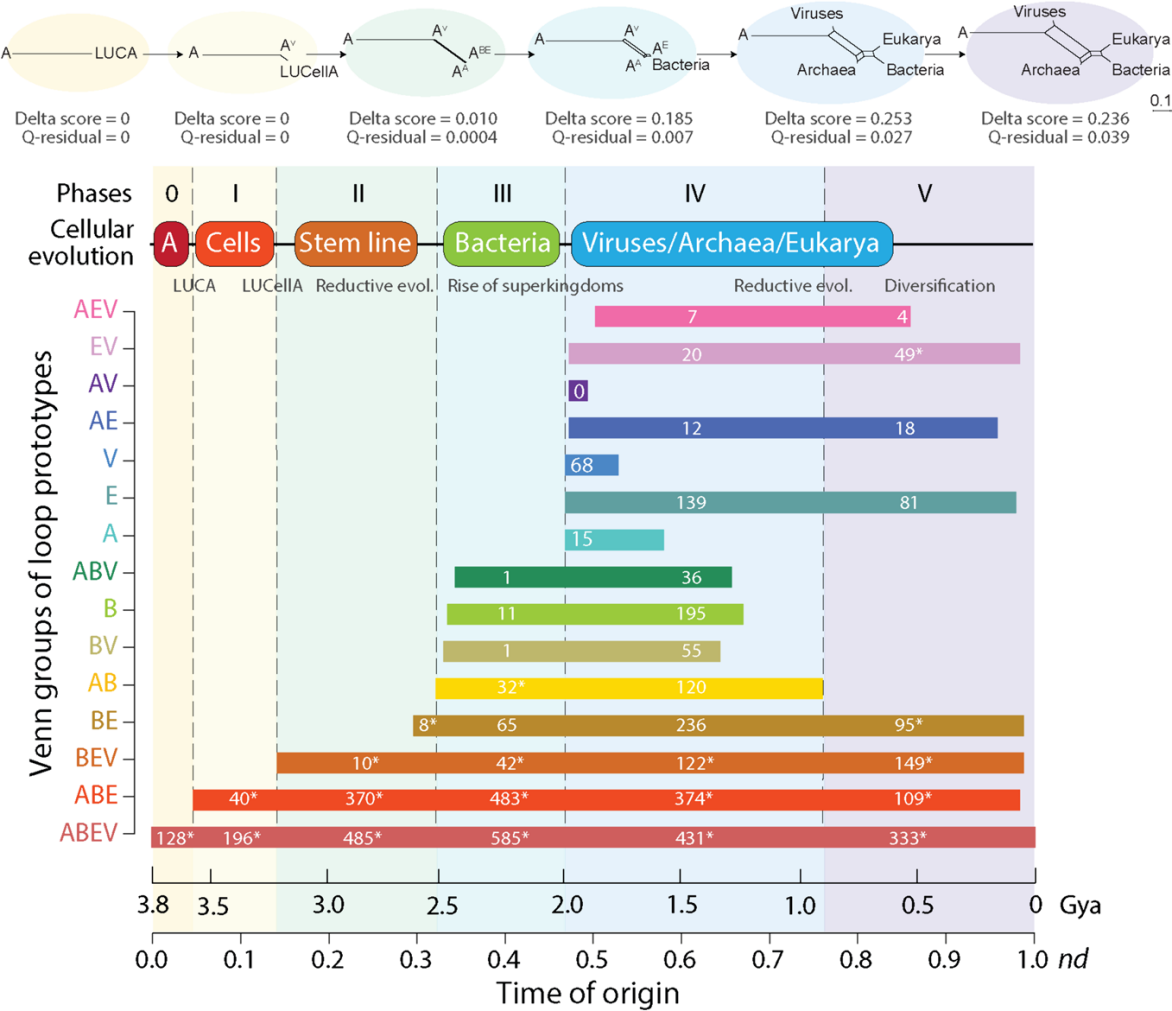
The evolutionary history of loop prototypes in the proteomes of cells and viruses. The chronology describes the gradual evolutionary appearance of non-modular prototypes belonging to the different Venn distribution groups. Numbers in bars indicate prototypes appearing in each evolutionary phase of the timeline and those labeled with asterisks larger values than those for corresponding fold families. A most likely chronology of cellular evolution inferred from Venn group distributions and phylogenetic reconstruction is shown on the top of bar plots. Ancestral cells (A) coalesce into a last universal common ancestor (LUCA), which then diversifies into a last universal cellular ancestor (LUCellA), a stem line of descent, ancestors of viruses (A^V^), Archaea (A^A^), Bacteria and Eukarya (A^AB^), and Eukarya (A^E^), and finally to modern diversified lineages of Archaea, Bacteria, Eukarya, and viruses. A series of phylogenetic networks implemented with the NeighborNet algorithm and uncorrected-P distances in SplitsTree4 (5 taxa; 15 characters weighted by prototype counts) confirms the early evolutionary rise of viruses and the evolutionary progression. Delta score calculations evaluate the amount of vertical phylogenetic signal present in the data. A score of 0 implies a fully bifurcating tree. A score of 1 implies a fully reticulated network

Intuitions inferred from prototype distributions in Venn diagrams, which were merely descriptive, were confirmed by tracing the times of origin of prototypes that were unique or shared among supergroups. The chronology described the progressive accretion of prototypes in the structural domains of the rising protein world along six clearly defined evolutionary phases, with Phase 0 being the most ancient (Fig. 4):

- *Phase 0: Communal world (3.8–3.6 Gya).* As expected for a system diversifying by vertical descent, this initial phase holds the first 128 prototypes to appear in evolution that are common to all cellular organisms and viruses and were part of the universal ABEV group. The oldest prototype of this phase was DS.EH.6.17.1, which we used in Fig. 3A to illustrate the ArchDB classification scheme. The prototype has β-strand and α-helical structures bracing a 6-residue aperiodic region with Ramachandran consensus *bb{eppvag}aa* (*see* Hollingsworth and Karplus 2010 for Ramachandran plot interpretations), which defines the P-loop of the oldest P-loop containing nucleoside triphosphate hydrolase fold (c.37). The rest of the prototypes populate the most ancient domain families (Caetano-Anollés et al. 2012), many of which harbor enzymatic activities linked to membranes, aminoacyl-tRNA synthetases responsible for the specificities of the genetic code and translation factors. The end of this phase defined LUCA, the common ancestor of cellular organisms and viruses. This common ancestor of the living world contained membrane-associated proteins and was therefore cellular in nature. In particular, the phase ended with the accumulation of 29 prototypes of families harboring intracellular and regulatory functions involved in inorganic ion metabolism and transport, small molecule binding, and signal transduction. Thus, the 128 prototypes of this phase represent the building blocks of a ‘pangenome’ and/or ‘panproteome’ harboring the structures and functions of a primordial communal cellular world pushing the establishment of a stable internal cellular environment (Caetano-Anollés et al. 2012).
- *Phase I: Rise of viral ancestors (3.6–3.2 Gya).* The second oldest period is characterized by two groups of prototypes, 196 universal (ABEV) and 40 shared by all superkingdoms (ABE) but not viruses. This indicates building blocks in this phase made up novelties that accumulated in two ‘stem lines’ of descent. These stem lines split LUCA in two cellular groups by reductive evolution: one grew to produce the modern cellular world and the other shrank to produce ancestral lineages that would later give rise to viruses. This phase began with the loss (less likely) or lack of gain of 10 prototypes belonging to the ABE category. Eight prototypes of this set were associated with the ‘thiolase-related’ family (c.95.1.1) holding the α-β-α-layered thiolase-like fold involved in numerous metabolic biosynthetic pathways, including those of fatty acid, steroid, and polyketide synthesis. Two prototypes (DS.GE.3.4.2 and DS.HE.7.2.1) were associated with the ‘acetyl-coA synthetase-like’ family (e.23.1.1) involved in acetate metabolism and entry points to a reverse citric acid cycle believed to be central to prebiotic chemistry (Caetano-Anollés and Caetano-Anollés 2024). Most accumulating prototypes participated in making up ribosomal and cell adhesion proteins together with proteins involved in core metabolic processes. This phase ended with a cellular ancestor (LUCellA) that gave rise to all cellular diversity. Reconstruction of minimal and maximal domain repertoires of LUCellA defined at SCOP fold superfamily (FSF) level revealed it had advanced metabolic capabilities, including nucleotide metabolic enzymes, pathways of biosynthesis of membrane *sn1,2* glycerol ester and ether lipids, and a primordial translation apparatus that included a biosynthetically active ribosome (Kim and Caetano-Anollés 2011). The 386 prototypes that were present at the end of this phase support these previous findings and confirm LUCellA was a functionally complex cellular entity (Nasir and Caetano-Anollés 2015).
- *Phase II: Birth of ancestors of Archaea (3.2–2.5 Gya).* The initial accumulation of 18 prototypes shared by Bacteria and Eukarya belonging to the BEV and BE groups marked the start of this phase. The first two prototypes of this set (DS.HH.1.1.58 and DS.HG.4.51.1) were associated with the ‘sigma2 domain of RNA polymerase sigma factors’ family (a.177.1.1). The appearance of these prototypes signaled reductive loss in primordial ancestors of Archaea and the rise of a cellular stem line common to Bacteria and Eukarya, superkingdoms that share a same membrane phospholipid makeup. The vast majority of prototypes of this phase however belonged to the ABEV (485) and ABE (370) groups, suggesting the rising stem lines were off-shoot grades rather than major diversifying lines.
- *Phase III: Diversified Bacteria (2.5–2.0 Gya).* The appearance of one prototype shared by Bacteria and viruses (the BV group), 11 prototypes specific to Bacteria (B), and 33 prototypes shared by Archaea and Bacteria (AB and ABV) revealed a slow accumulation of the first superkingdom-specific structures and the emergence of Bacteria. The first 3 prototypes (DS.EH.4.124.1, DS.GE.5.8.1, and DS.HE.2.2.7) belonged to the AB group and were associated with the ‘Ykg-like’ family (c.124.1.7) of transferases. The first bacteria-specific prototypes were linked to the ‘sporulation-related repeat’ family (d.58.52.1), which is a widely common peptidoglycan-binding domain of bacteria, and the ‘GlnE-like domain’ family (d.218.1.9) with nucleotide metabolism and transport functions. Prototype appearance strongly suggests the onset of a diversified cellular world. Note that while the archaeal stem line splits earlier it was Bacteria that first diversified to produce bacterial-specific prototypes and domain families. Bacterial diversification during this phase is supported by microfossil evidence, which culminated with the identification of an unambiguous cyanobacterial fossil record 1.89–1.84 Gya (Demoulin et al. 2019). While detection of 2-methylhopanoid biomarkers 2.7 Gya (Summons et al. 1999) constitute perhaps signatures of other anoxygenic phototrophs (Rashby et al. 2007), there is credible evidence supporting filamentous *Siphonophycus* cyanobacterial microfossils 2.5 Gya, *Oscillatoriopsis* microfossils 2.2 Gya, and *Archaeoellipsoides* microfossils 2.1 Gya (Demoulin et al. 2019). A link of emerging cyanobacteria to the Great Oxygenation Event (GOE) that occurred 2.45 Gya during the start of this phase remains contentious. However, the earliest oxygen-utilizing domain, the ‘PNP-oxidase like’ family (b.45.1.1), appeared 2.9 Gya (*nd* = 0.210) in Phase II together with its 4 associated prototypes (DS.EH.2.5.4, DS.EH.3.5.3, DS. EH.3.5.6, and DS.HE.2.2.13). These structures made synthases that produced pyridoxal 5’-phosphate or pyridoxal from an oxygen source catalyzed by the metal-storing ‘ferritin’ family (a.25.1.1) and its 3 associated prototypes (DS.HH.5.61.1, DS.HH.8.3.1, DS.HH.1.1.42) that appeared ~ 3 Gya (*nd* = 0.180). This family of structures likely materialized before the GOE in a Mn catalase that generated oxygen as side product of hydrogen peroxide detoxification in Pongola Supergroup glacial meltwaters (Kim et al. 2012).
- *Phase IV: Rise of diversified superkingdoms and viruses (2.0–0.9 Gya):* The appearance of prototypes specific to Archaea, Bacteria, Eukarya, and modern viruses in this phase marked the rise of diversified lineages. The largest number of superkingdom-specific prototypes corresponded to the B, AB, and E groups suggesting the significant role of loops in determining molecular functions of emerging bacterial and eukaryotic organisms. In addition, a total of 12 and 7 prototypes belonging to the AE and AEV groups suggest horizontal exchanges between the rising archaeal and eukaryotic organisms. Note that the late appearance of all of these Venn groups is only compatible with the late emergence and concurrent diversification of cellular lineages and modern viruses. This conclusion is reinforced by virus-specific prototypes (V) linked to capsid and coat folds that are necessary for viral infection (Nasir and Caetano-Anollés 2015; Mughal et al. 2020). Thus, parasitism appears to be an evolutionarily late development. This is an expected outcome. Pathogenesis implies differential recognition of hosts, which is only possible if hosts are operating as diversified lineages.
- *Phase V: Rise of Eukarya (0.9 Gya to the present):* This last phase is enriched in prototypes belonging to the E and EV groups, suggesting a major diversification push occurring in Eukarya. A total of 24 prototypes belonging to the AE and AEV again suggested coevolution between Eukarya and Archaea, especially involving informational proteins (e.g., RNA and DNA polymerase). Notably, a number of Venn groups were completely missing in this phase, especially those involving Bacteria (AB, ABV, B, and BV). These bacterial-specific prototypes were for some reason being only channeled via domain families shared with Eukarya (BE and BEV groups) during this late evolutionary stage. Prototypes belonging to the 374 families that involved multicellularity unfolded between the appearance of the 4 prototypes of the ‘Integrin A (or I) domain’ family (c.62.1.1) 3.2 Gya and the present. Most of them emerged during Phases IV and V, showing these two last phases carried most prototypes linked to the development of multicellular organisms. To illustrate, out of 99 prototypes linked to 37 families harboring the ‘cell adhesion’ functional category, 20 prototypes mapping to 13 families appeared in Phase IV and 66 prototypes mapping to 19 families appeared in Phase V.

It is noteworthy that similar Venn group distribution patterns and phases of accumulation were obtained when studying the accretion of structural domains defined at different levels of SCOP classification (Caetano-Anollés 2005; Wang et al. 2007; Kim and Caetano-Anollés 2011, 2012, 2014; Nasir and Caetano-Anollés 2015; Staley and Caetano-Anollés 2018; Mughal et al. 2020), accretion of CATH topologies and homologous superfamily domain structures (Bukhari and Caetano-Anollés 2013), and accretion of molecular functions at different levels of GO database classification (Kim and Caetano-Anollés 2010; Koç and Caetano-Anollés 2017), and from data that included or excluded viruses (compare studies of domains defined at family level: Staley and Caetano-Anollés 2018; Mughal et al. 2020). Such congruence of evolutionary statements derived from different features of structure and function and in different studies is striking.

The present analysis of prototype building blocks of domains however uncovered some significant evolutionary patterns in the data. First, prototypes of the most basal Venn groups were overrepresented when compared to domain families belonging to those same groups (numbers labeled with asterisks in Fig. 4). Second, the numbers of prototypes appearing in each phase were substantially larger than families during the first 4 phases (ratios ranging 6.4–1.5), mostly driven by basal Venn groups (Table 1). Thus, a clear decreasing pattern of non-modular prototype representation unfolded in evolution. Since prototypes are building blocks that compete in making domain structures, the trend of decreasing ratios probably reflects the massive recruitment of modular prototypes that started to unfold half way along the evolutionary chronology (Fig. 3B). Third, the gradual evolutionary accumulation of prototypes occurring throughout the six evolutionary phases suggest prototype novelties are permanently being generated and used throughout the timeline. Finally, the overrepresentation of basal Venn groups strongly supports pervasive recruitment operating across all emerging stem lines and lineages of the evolutionary progression.

**Table 1:** Comparison of non-modular loop prototypes and domain families developing along the six phases of the evolutionary chronology

	Evolutionary phases					
	0	I	II	III	IV	V
Prototypes	128	236	873	1220	1830	838
Domains	20	59	323	818	2115	557
Ratio	6.40	4.00	2.70	1.49	0.87	1.50

**Fig. 5:**
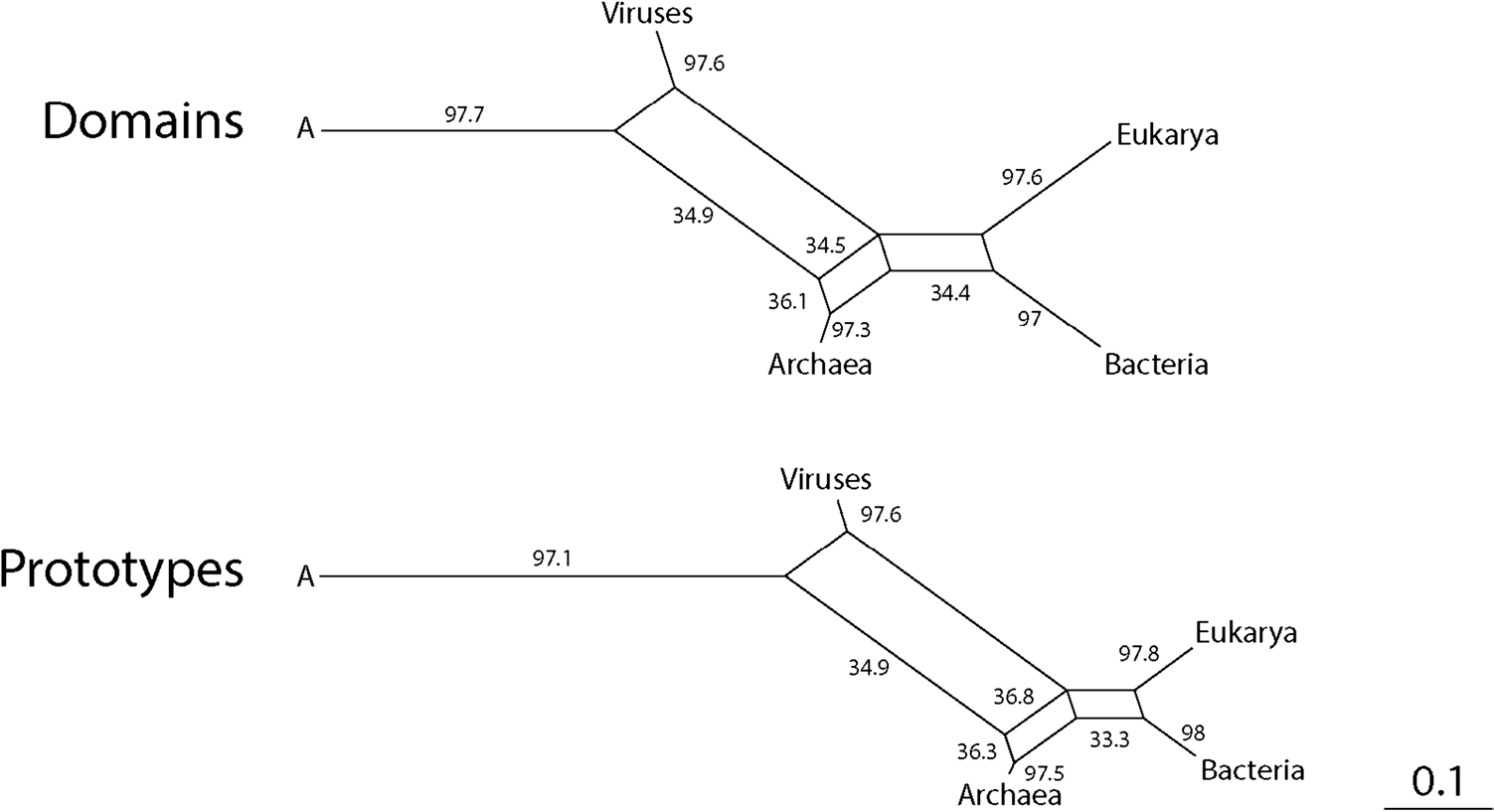
Phylogenetic networks describing the evolution of superkingdoms and viruses were reconstructed from Venn distribution data for structural domains and loop prototypes. The networks were implemented with the NeighborNet algorithm using uncorrected-P distances in SplitsTree4 (5 taxa; 15 characters weighted by prototype counts). Bootstrap support values (%) are given for individual edges following a bootstrap analysis with 2000 replicates. Delta score calculations for domains (Delta score = 0.227, Q-residual score = 0.038) and prototypes (Delta score = 0.236, Q-residual score = 0.039) show comparable and significant vertical signatures in the data

A most parsimonious chronology of cellular evolution can be inferred from Venn group distribution data (top chronology of Fig. 4). In this chronology, ancestral cells (A) first coalesced into LUCA, which then generated LUCellA and an ancestral stem line leading to modern viruses (A^V^) by forces of reductive evolution. LUCellA then produced ancestors of Archaea (A^A^) and of Bacteria and Eukarya (A^AB^), which then diversified into Bacteria and ancestors of Eukarya (A^E^). Finally, the last two phases resulted in a modern diversified world of organismal lineages reflecting all superkingdoms and viruses. A series of phylogenetic networks built from Venn distribution patterns using both the Neighbor-Net algorithm (top of Fig. 4) or the split decomposition method (not shown) in SplitsTree4 (Huson and Bryant 2006) confirmed the proposed evolutionary progression. In these reconstructions, Delta score calculations revealed significant vertical phylogenetic signal in the data, especially during the first evolutionary phases. This supports the stem line concept. A comparison of phylogenetic networks reconstructed at the end of Phase V (the present) with bootstrap support levels for edges showed equal levels of reticulation for prototype and domain family evolution and identical phylogenetic network topologies (Fig. 5). Thus, chronologies of prototype and domain families reflect similar history.

## Evolution of Loop Types and the Birth of Domain Structural Cores

**Fig. 6:**
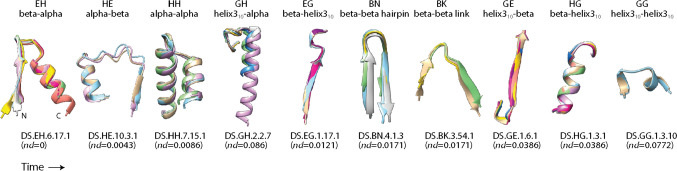
The most ancient non-modular loop prototypes belonging to the ten loop types of the ArchDB classification. Prototypes are illustrated with structural alignments of no more than 10 loop structural entries using Chimera and indexed with times of origin (*nd*)

**Fig. 7:**
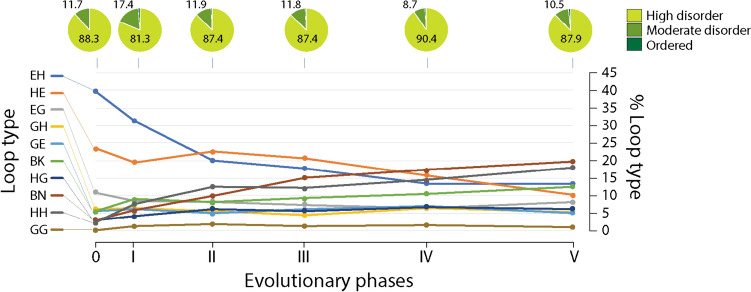
Evolution of ArchDB loop type and disorder levels along the phases of the evolutionary timeline. Note the highly enriched helix and strand bracing structures during the first few phases and the gradual rise of the rest of types of bracing structures

**Fig. 8:**
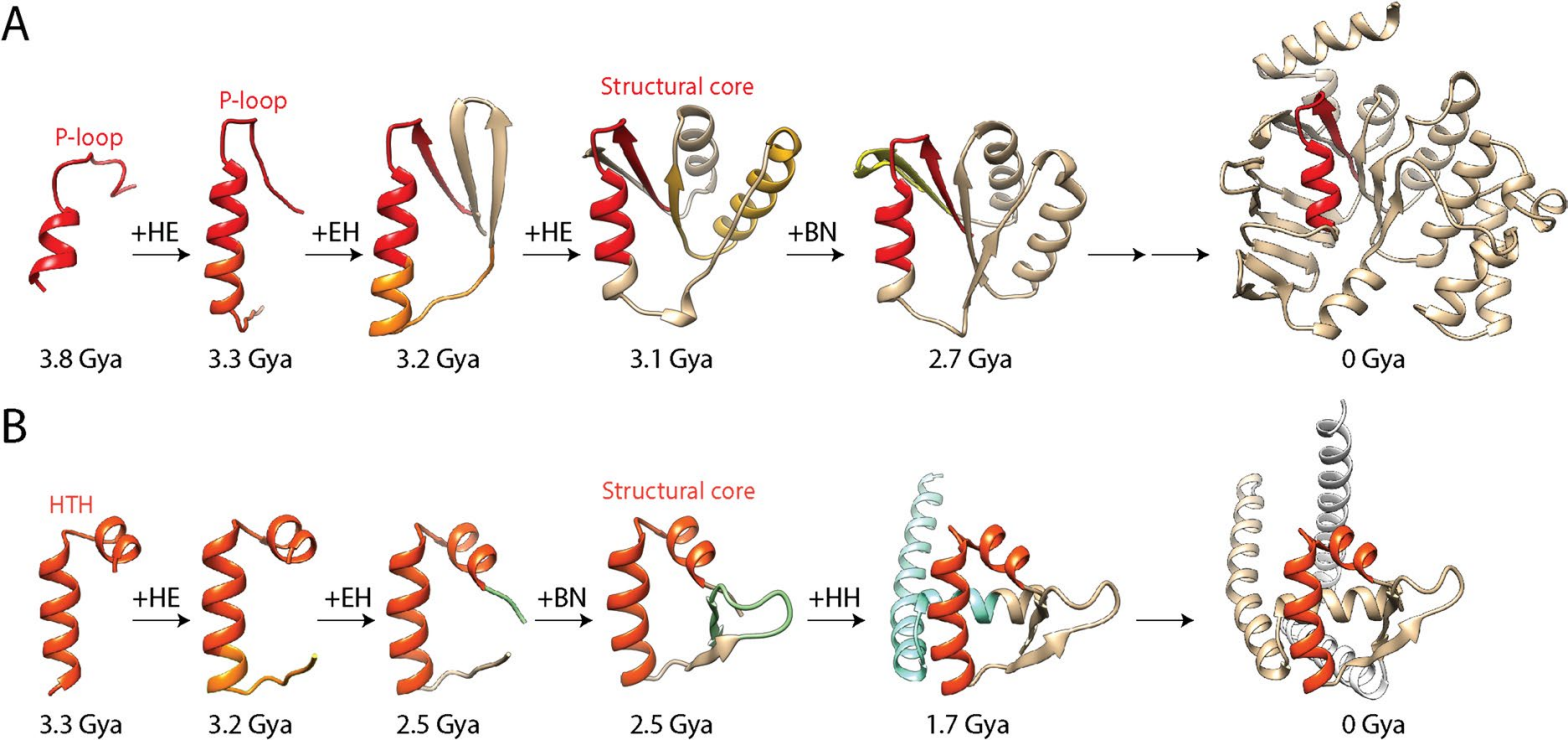
Modeling the birth of structural domains from loop prototypes with AlphaFold2. A time ordered series of growing atomic structures of the P-loop containing ATP-binding domain (**A**) and helix-turn-helix (HTH) containing winged helix domain (**B**) modeled directly from their sequences. Each step of the timeline adds a new loop type to the fold makeup, which is labeled with ArchDB loop-type nomenclature. Fully formed structural cores are indicated in the timeline and can be downloaded from the ModelArchive structural repository under accessions *ma-gca-proto-04* and *ma-gca-proto-18* (https://doi.org/10.5452/ma-gca-proto). Modified from Aziz et al. (2023)

The chronology of prototypes revealed that all possible bracing supersecondary structures typical of loop types made their appearance very early and gradually in the protein world. They did so earlier than 3.5 Gya in urancestors, 9 types in LUCA, and one in LUCellA. Figure 6 shows atomic models describing the structure of the most ancient prototypes belonging to the 10 loop-type categories. Tracing the relative abundance of these types of building blocks along the phases of the evolutionary chronology showed that they did not accumulate at equal rates (Fig. 7). A clear pattern of decrease of loop types with α-helix and β-strand bracing structures (EH and HE types) was offset by a gradual increase of all other loop types, especially the α-helix and α-helix (HH) and β-strand and β-strand (BN) bracing motifs. At the end of Phase V (the present), the popularity of HH and BN motifs completely replaced that of the EH and HE types. The early overrepresentation of the EH and HE types has probable roots in their ability to pack helical structures onto small sheets of β-strands, which are typical of the α-β-α-layered fold structures of the most ancient domain families (Caetano-Anollés et al. 2012). In fact, AlphaFold2 modeling showed these types of folded structures materialized relatively quickly by accretion of loop prototypes (Aziz et al. 2023). The central core of the oldest domain structure, the ‘ABC transporter ATPase domain-like’ family (c.37.1.12), which packs the helical bracing structure of its P-loop against an emergent sheet of three β-strands, unfolded during the lifetime of the stem line leading to LUCellA (Fig. 8A). In contrast, it took considerably longer to build the winged helical structure embedding the HTH motif of the quite ancient ‘MAR-type transcriptional regulator domain’ family (a.4.5.28) (Fig. 8B). We are currently extending the use of deep learning algorithmic implementations to model the birth of domains appearing throughout the timeline and understand how building blocks are used to construct complex fold structure.

**Fig. 9:**
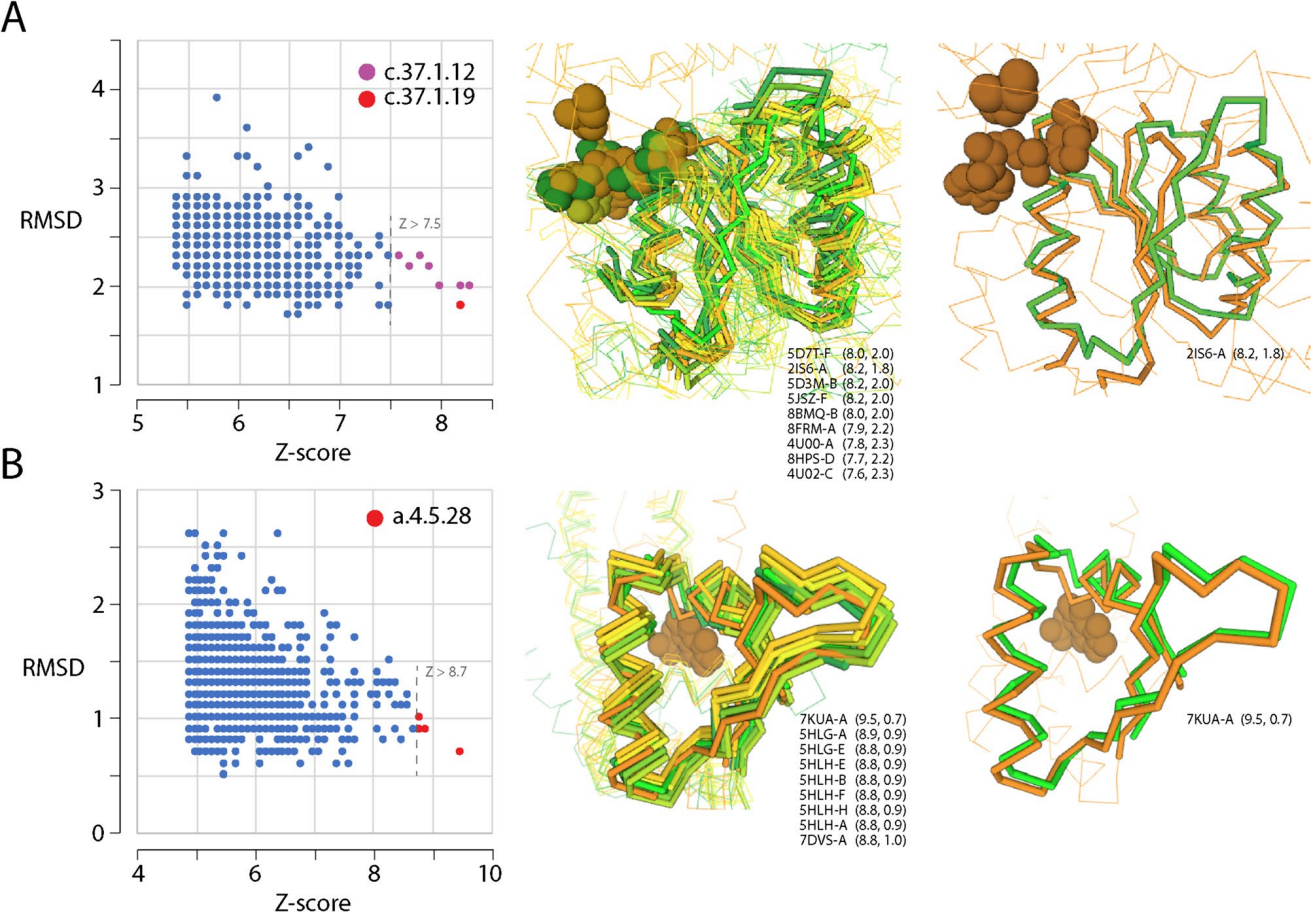
DALI structural neighborhoods and best structural alignments to structural core intermediates belonging to the P-loop containing ATP-binding domain (**A**) and helix-turn-helix (HTH) containing winged helix domain (**B**). RMSD versus Z-score plots illustrate neighborhoods of 1000 best structures extracted from 594,244 PDB chains (computed March 2, 2024) by submitting AlphaFold2 rank 1 PDB queries. Structural alignments of matches above a Z-score threshold (indicated in the plots and indexed with domain family SCOP identifiers) show a tight structural fit between the query (light green) and corresponding regions of PDB models (listed together with Z-score and RMSD values in parentheses). Alignments against the best Z-score matching structures, an UvrD helicase from *Escherichia coli* (PDB entry 2IS6-A) and a MarR transcriptional regulator from *Pseudomonas aeruginosa* (7KUA-A), are also provided

Since the important c.37.1.12 and a.4.5.28 evolving structures are responsible for the first primordial waves of functional innovation and recruitment, we determined if their central structural core intermediates that developed 3.1 and 2.5 Gya (Fig. 8) had close structural relatives in the extant world of proteins. To do so, we studied the structural neighborhoods of these two prior molecular states using the DALI server (Holm 2022). DALI is a fully automated non-hierarchical structural alignment comparison tool that produces summary lists of structural neighbors from a distance matrix. Structural similarities are expressed as root-mean-square deviation (RMSD) of rigid body structural superpositions. Z-scores describe length-rescaled distance matrix alignments that maximize one-to-one atomic correspondences between two structures with a weighted sum of similarities of intramolecular distances. They provide a measure of alignment length. Note that better and longer structural matches get lower RMSD and larger Z-score values, respectively. Figure 9 shows RMSD versus Z-score plots describing the structural neighborhoods of the structural cores. As expected, the closest structural neighbors had structures that matched those they would later produce, the folds of the c.37.1.12 and a.4.5.28 domain families. Surprisingly, however, the structural core of the oldest P-loop containing domain family had also one close match to a structural relative, the ‘tandem AAA-ATPase domain’ family (c.37.1.19) embodied in an unusual nucleic acid helicase (PDB entry 2IS6). This helicase has been shown to unwind double helices of nucleic acids one base pair at a time using a two-part power stroke driven by a combined wrench-and-inchworm mechanism (Lee and Yang 2006) (Fig. 9). Structural alignments to the closest P-loop containing ABC transporters (Fig. 9A) and HTH motif-containing transcriptional regulators (Fig. 9B) showed that the structural cores involved the central nucleic acid-binding functions typical of these domains families. This suggests that their ancestral molecular functions have been preserved in evolution. Two protein engineering studies are particularly significant in this respect. Small 55 residue-long P-loop–containing polypeptides were found to be catalytically active and capable of binding a range of ligands, including RNA and single-stranded DNA (Romero Romero et al. 2018). Similarly, a 40-residue polypeptide comprising just one P-loop element acted as a helicase separating and exchanging nucleic acid strands (Vyas et al. 2021). Both studies support the early nucleic acid-linked functionality of our molecular core intermediates. It is therefore remarkable that the structural core intermediates of the P-loop and HTH-containing domain structures converged to a common structure in which a central helix component of a nucleic acid-binding loop prototype was packed against a small β-sheet structure to facilitate hydrolase and helicase roles.

## Evolution of Loop Geometric Properties

**Fig. 10:**
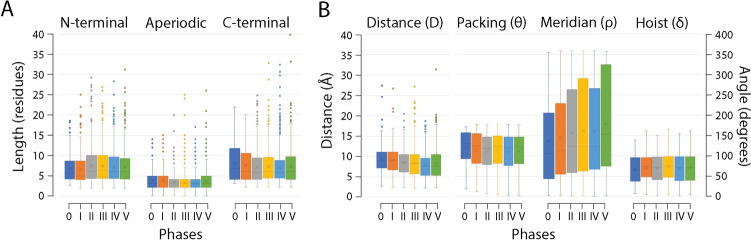
Evolution of structural and geometric properties of loop prototypes. (**A**) Box plots describe the distribution of the average lengths of the N-terminal and C-terminal bracing structures and the central aperiodic region of the loop prototypes along the evolutionary phases of the timeline. (**B**) Box plots describe the distribution of the average geometric properties of the distance between the boundaries of the aperiodic structure (D) and the packing, meridian, and hoist angles that modify the return of the polypeptide chain of the loop prototypes along the phases of the timeline

Extending initial evolutionary studies of the structural and geometric properties of prototypes (Mughal and Caetano-Anollés 2023) highlighted new patterns. The distribution of the average lengths of the N-terminal and C-terminal bracing structures and the central aperiodic region of the prototypes were quite constant throughout the evolutionary phases of the timeline (Fig. 10A). This shows that the size of building blocks is evolutionarily conserved. This is surprising since we previously found a Menzerath–Altmann’s law of domain organization in which larger molecular systems had smaller parts (Shahzad et al. 2015). Thus, lower-level modules appear to behave differently than higher-level ones. One observation is however noteworthy. During the first two phases, which embody urancestors, the C-terminal bracing structures of the prototypes appeared longer than the N-terminal counterparts. This asymmetry is puzzling. It could be linked to preferences in how polypeptide chains were being extended in these early phases of protein evolution.

We also compared the distribution of the average geometric properties of prototypes, including the distance (D) between the boundaries of the aperiodic structure and the packing, meridian, and hoist angles that modify the return of the polypeptide chain (Fig. 10B). Remarkably, trends toward the evolutionary reduction of D and expansion of meridian angles were evident in the timeline, perhaps suggesting that loop structures were becoming more compact and combinable. Reduction of D bring bracing structures closer together, while larger meridian angles allow for wider polypeptide return conformations. An analysis of meridian angles of individual loop types (not shown) shows that the distribution medians of the BN (334.4°) and HH (149.2°) types were significantly larger than those of the EH (141.5°) and HE (109.8°) types. Thus, the upward evolutionary trend of meridian angles is explained by the gradual replacement of EH and HE types by BN and HH types we observed occurred in evolution (Fig. 10).

## The Loop Repertoires of the Common Ancestors of Life

**Fig. 11:**
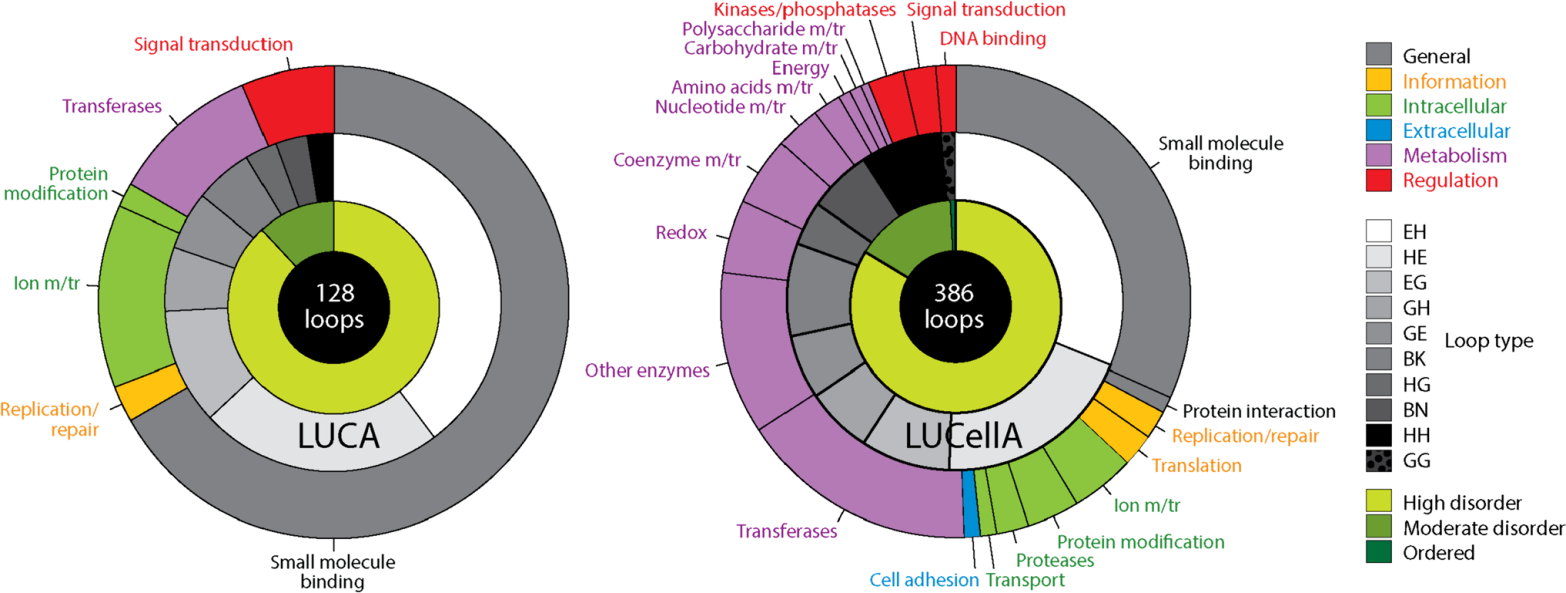
The non-modular loop repertoires of the last universal common ancestor (LUCA) and the last universal cellular ancestor (LUCellA) of life. Urancestral prototype annotations with ArchDB loop type and disorder levels (internal ring charts) show that prototypes are highly enriched in helix and strand bracing structures and are highly disordered. Functional annotations (external ring chart) using *superfamily* classification of 7 major and 50 minor functional categories (https://supfam.mrc-lmb.cam.ac.uk/SUPERFAMILY/function.html) reveal functionally complex urancestors. Major functional categories (definitions): General (general and multiple functions; interactions with proteins/ions/lipids/small molecules); Information (storage; maintenance of the genetic code; DNA replication/repair; general transcription/translation); Intracellular processes (cell motility/division; cell death; intra-cellular transport; secretion); extracellular processes (intercellular and extracellular processes, e.g., cell adhesion; organismal processes, e.g., blood clotting, immune system); Metabolism (anabolic and catabolic processes; cell maintenance/homeostasis; secondary metabolism); Regulation (regulation of gene expression and protein activity; information processing in response to environmental input; signal transduction; general regulatory or receptor activity); and Other/Unknown (unknown function, viral proteins/toxins). m/tr = metabolism and transport

The non-modular prototypes of LUCA and LUCellA are particularly insightful and can be explicitly derived from the chronology. Multilevel ring charts describing annotated molecular functions, bracing secondary structures of the protein loops (loop types), and levels of intrinsic disorder showed that both urancestors were functionally and structurally complex (Fig. 11).

Annotations of prototypes with molecular functions of the *superfamily* domain classification system (Vogel et al. 2004, 2005) showed urancestors were functionally complex. Of the 7 major and 50 minor (more detailed) functional categories of the classification system, LUCA and LUCellA showed 5 major/6 minor and 6 major/21 minor categories, respectively. These sets represent significant swaths of molecular functions embedded within relatively simple structural repertoires. These results are unsurprising. A number of early studies had already uncovered a simple yet relatively complex urancestral functionome, some of which can be found indexed in the LUCApedia database (Goldman et al. 2013). One particular study applied advanced evolutionary bioinformatics tools and an iterative approach of CSR reconstruction to identify most parsimoniously a lower and upper bound of the FSF domain repertoire of LUCellA (Kim and Caetano-Anollés 2011). These bounds defined conservative limits to the diversity of domains and functions. The lower bound had 70 FSF domain structures, orders of magnitude below that of extant organisms. However, these FSFs reflected advanced metabolic capabilities, including nucleotide metabolic enzymes, pathways of biosynthesis of membrane *sn1,2* glycerol ester and ether fatty acid lipids, and a primordial translation apparatus that included aminoacyl-tRNA-synthetases (aaRSs), regulatory factors, and a ribosome with protein biosynthetic capabilities. The repertoire was especially rich in hydrolases and transferases and numerous membrane proteins that controlled transport of small and large molecules in and out of cells thanks to their small molecule-binding abilities. It is also likely that the urancestor of cells had structures necessary for cellular organization such as filaments and primordial cytoskeletal structures, but lacked processes of extracellular communication (other than cell adhesion). Most notably, the study revealed it did not contain the catalytic domains of ribonucleotide reductase enzymes necessary to produce deoxyribonucleotides, the ‘ferritin-like’ (a.25.1), the ‘N-terminal domain of cbl (N-cbl)’ (a.48.1) and the ‘PFL-like glycyl radical enzyme domain’ (c.7.1) FSF structures. Consequently, LUCellA did not store genetic information in DNA. Instead, it stored genetic information in RNA molecules but without explicit processes of transcription. The loop prototype repertoire of LUCellA described in Fig. 11 supports each and every one of these published findings. In fact, only one (signal transduction) out of the 21 minor functional categories identified in LUCellA was absent in the 20 minimum set identified by Kim and Caetano-Anollés (2011). The match is remarkable considering that prototypes were mapped to domain family and not superfamily structures and that the present study included viruses. The absence of prototypes associated with catalytic domains of ribonucleotide reductase enzymes in LUCellA and their later appearance also supports the lack of a DNA genome. For example, the three prototypes associated with the ‘ferritin’ family (a.25.1.1) mentioned above appeared in Phase II and one (DS.HH.4.50.1) associated with the ‘ribonucleotide reductase-like’ family (a.25.1.2) appeared later in Phase IV. Similarly, 15 prototypes associated with families of ribonucleotide reductase, including the oldest ‘R1 subunit of ribonucleotide reductase, C-terminal domain’ family (c.7.1.2), appeared in Phase IV.

We also examined ArchDB loop types present in the repertoires of the urancestors (Fig. 11). Recall that a prototype describes the geometry and conformation of a loop structure that is part of a protein domain. These properties define the type of return of the polypeptide chain operating in that molecule. Consequently, a larger diversity of prototypes implies a larger collection of building blocks and a larger ‘vocabulary’ for solving the protein folding problem (its ‘origami’). LUCA contained 9 of the 10 loop types that are possible while LUCellA contained all 10 of them. This already suggests folding designs were quite advanced at these very early stages of evolution. Note however that the vast majority of prototypes of urancestors had α-helix and β-strand bracing structures (EH and HE types), with LUCA being particularly rich in these loop arrangements. These prototypes are needed to make α/β and α + β domains, which are known to be the most ancient (Caetano-Anollés and Caetano-Anollés 2003). Also note the significant underrepresentation of α-helix and α-helix (HH) and β-strand and β-strand (BN) bracing arrangements that are needed to build all-α and all-β domains, respectively, which are more evolutionarily derived (Caetano-Anollés and Caetano-Anollés 2003). This over- and underrepresentation is therefore an expected outcome.

Finally, the study of intrinsic disorder of loop prototypes of urancestors revealed they were highly disordered (Fig. 11). Only 11.7% of prototypes showed moderate disorder in LUCA and only 14.5% and 0.8% showed moderate disorder or were ordered in LUCellA, respectively. These patterns support the ancestral nature of intrinsic disorder in protein structure (Mughal and Caetano-Anollés 2023).

## The Problem of Ancestors

German zoologist Ernst Haeckel (1966) depicted at the base of his beautiful Tree of Life (ToL) illustrations a ‘*radix communis organismorum,’* the common root of all organisms. Ever since then, LUCA has been in the imagination of scientists and the public alike. In ToL representations, LUCA is often placed at the base of phylogenetic tree reconstructions that have been rooted using various implementations or more commonly with argumentative ad hoc assumptions (Caetano-Anollés et al. 2018). CSR studies that were more ambitious attempted to build vectors of phylogenetic characters describing this urancestor (e.g., using iterative approaches; Kim and Caetano-Anollés 2011). Here, phylogenetic characters selected from extant data are assumed suitable to make inferences about ancient features that were present in LUCA. This in itself is problematic because life expresses layers of organization that often follow hierarchical relationships, many of which may have not materialized during the life of urancestors. For example, when examining the fundamental role of intrinsic disorder in loop prototype functionality we found evolutionary constraints percolating from higher to lower levels of protein organization (Mughal and Caetano-Anollés 2023). These constraints resulted in trade-offs between flexibility and rigidity that impacted prototype structure and geometry. Thus, constraints percolating from advanced domain organization in evolution could soften or harden trade-offs and change protein functionality.

LUCA has been generally considered an authentic ancestor from which all modern organismal lineages were generated: an “Adam” of all life. However, the gradual appearance of loop prototypes and structural domains suggests this may represent a misconception. The common ancestor of diversified life embodied a transition between prior cellular states of a communal type and modern diversifying counterparts in the form of organismal lineages. This sort of transition was already anticipated by Carl R. Woese. Using a genetic annealing model, Woese proposed a communal world in which ‘genetic temperatures’ were high (i.e., their evolutionary ‘tempo’ increased rates of change), cellular entities were simple, genetic processing was inaccurate, and mutation rates and horizontal genetic transfer were rampant (Woese 1998). Woese regarded this urancestor as a conglomerate of cells in search of cohesion: *“The ancestor cannot have been a particular organism, a single organismal lineage. It was communal, a loosely knit, diverse conglomeration of primitive cells that evolved as a unit, and it eventually developed to a stage where it broke into several distinct communities, which in their turn become the three primary lines of descent. The primary lines, however, were not conventional lineages. Each represented a progressive consolidation of the corresponding community into a smaller number of more complex cell types, which ultimately developed into the ancestor(s) of that organismal domain. The universal ancestor is not an entity, not a thing. It is a process characteristic of a particular evolutionary stage.”* (Woese 1998). Under this ‘processual’ view, LUCA was not the endpoint of an emergent lineage. Instead, it was a ‘stem line,’ a propagating series of pluripotent cellular entities, perhaps a ‘megaorganism’ producing poorly-defined off-shoots of self (perhaps akin to evolutionary grades) in the changing environments of a primordial planetary landscape (Caetano-Anollés et al. 2014). The phylogenomic timeline of loop prototypes described in Fig. 4 provides strong support to this evolutionary model. For example, only relatively few prototypes define the rise of lineages in Phases III–V, while a steady dynamic underground involving numerous prototypes was pervasively generated throughout the timeline.

**Fig. 12:**
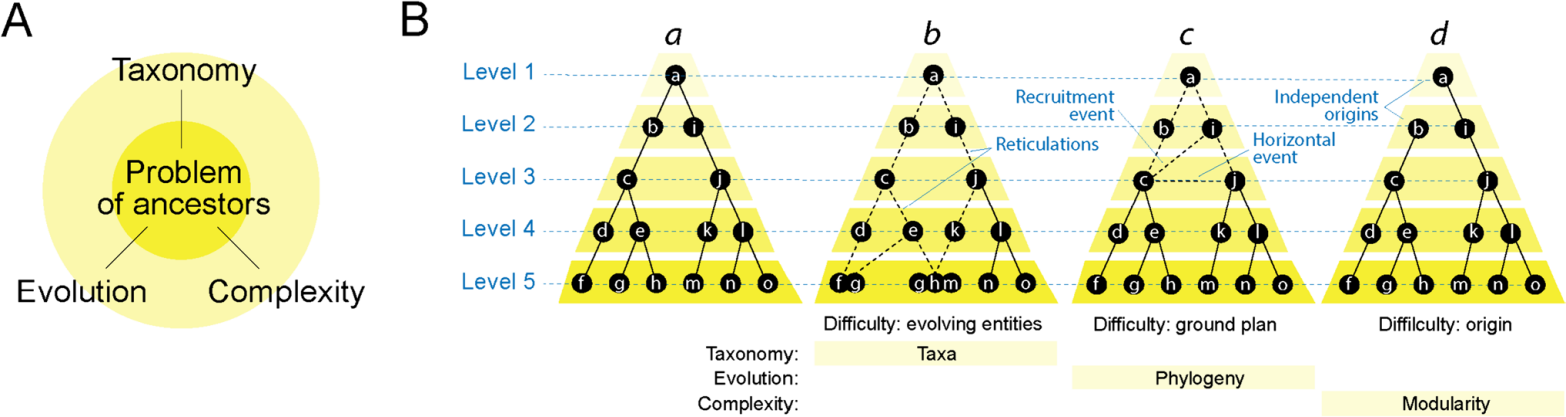
The problem of ancestors. (**A**) The problem of ancestors is central to the fields of taxonomy, evolution, and complexity. (**B**) Given levels of organization and a ground plan that describes how evolving entities originate and then generate/traverse those levels, a pyramid structure with an embedded network (*a*), often described with a ‘subsumption’ (specification) hierarchy, is challenged by three main difficulties: definition of the evolving entities (taxa) (*b*), the ground plan that describes their evolution (phylogeny) (*c*), and the origin of those entities, which often behave as modules (modularity) (*d*)

A number of difficulties plague our interpretation of ancestors and challenge retrodiction. We will call these difficulties the *‘problem of ancestors’* and show it impacts the fields of taxonomy, evolution, and complexity (Fig. 12A). In order to provide structure to reality and an integrative philosophical framework, biologists have used the concept of ‘level of organization’ to organize phenomena emerging in evolutionary time from other phenomena occurring at a different level. These phenomena relate to biological entities, structures, and processes. Bunge (1960) however provided semantic clarification for ontological speculation by assigning 9 different meanings to levels: degree, degree of complexity, degree of analytical depth, emergent whole, poistem, rank, layer, rooted layer, and grade. When levels are considered ‘degrees,’ they represent grades in a static scale or stages in a process. Three examples are relevant to our discussion of ancestors, levels of abundance of domains or prototypes (an extensive property), levels of *superfamily* functional classification (a processual property), and levels of abstraction in the SCOP classification hierarchy (a complexity property). When levels are considered ‘emergent wholes’ their members become mereological entities describing integration and diversity, self-contained units like prototypes, or cells in which parts are brought together through harmonious mutual action (integration through interaction). These emergent wholes are often considered ‘poistems’ when systems express interrelated qualities or variables (e.g., Venn groups of prototypes), ‘ranks’ when systems are viewed as hierarchies, grades on a hierarchy, or staircase pyramids of dependencies (e.g., taxonomic classification of species), ‘layers’ when systems are placed in stratigraphic arrangement according to the order of emergence in time (e.g., geological strata, time of origin of prototypes), or ‘rooted layers’ when systems are considered emergent but rooted at a lower level (e.g., timetrees). Finally levels can represent ‘grades,’ linear or non-linear relationships of order that are part of an evolutionary progression and have emerged in time from lower or higher pre-existing levels. In Fig. 12B, we use a staircase pyramid embedding a nonreticulated network representation (pyramid *a*) to describe biological levels and difficulties of interpretation in taxonomy, evolution, and complexity.

(i) *Taxonomy:* From a taxonomy perspective, taxonomic units (taxa) are organized with a scheme of hierarchical classification with levels representing ranks in linear sequence. In this multi-level system lower ranks with highly populated members depend on less populated higher ‘deeper’ ranks, different ranks lack common members, and the lowest rank generally embeds the units of classification. This Linnaean-like system uses, for example, a tree paradigm to classify organisms and viruses in groups reflecting ‘natural’ evolutionary relationships (e.g., Hugenholtz et al. 2021; Caetano-Anollés et al. 2023) or organizes the structural domains of proteins into families, superfamilies, folds, and classes according to sequence and structural similarities (e.g., Murzin et al. 1995; Fox et al. 2014). One difficulty of these classification schemes, which seek to become evolutionary, is that members of ranks can have blurry boundaries or can integrate with other members to introduce reticulations. This challenges the hierarchical structure of the ranked system (pyramid *b*). Examples of this problem are holobionts, organismal communities organized around individual hosts that exhibit synergistic phenotypes, have integrated ‘hologenome’ systems, and evolve in coordination, challenging the concept of ‘individuality’ and ‘organismality’ (Queller and Strassmann 2009; Gilbert et al. 2012). Holobionts such as microbiomes and coral communities introduce co-evolutionary relationships that challenge taxonomic units and the identity of ranks and complicate mapping relationships between them. If ranks describe levels organized by common ancestors (e.g., superkingdom Eukarya defined by an ancestor of all eukaryotes), then evolutionarily deep holobiont relationships that blur taxonomic boundaries challenge the identity of ancestors by making them resemble Woesian communities rather than endpoints. Similarly, and at the molecular level, not all fold structures fold into discrete entities in the space of possible folds making them ‘gregarious’ (Harrison et al. 2002), while structural similarities may also unfold at higher levels of fold classification (Edwards and Deane 2015; Mura et al. 2019). Here, a taxonomical reality challenges the demands of evolution and complexity, complicating evolutionary classification but also the retrodictive method.
(ii) *Evolution:* From an evolutionary perspective, phylogenies establish the ground plan needed to fulfill the *singularity* axiom. By necessity and because of computational limitations, the ground plan is an unrooted phylogenetic tree with no reticulations. However, the existence of evolutionary forces of horizontal exchange, including lateral transfer, hybridization, recombination, reassortment, fusion, endosymbiosis, recruitment, de novo creation, and other entanglements is pervasive. This fact demands that reticulations be accounted for by building phylogenetic networks (Caetano-Anollés et al. 2023) and that a more pluralistic and network-driven ‘processual’ ontology be formalized (Bapteste and Dupré 2013). Recruitments are particularly pervasive and problematic (Caetano-Anollés et al. 2022). They often cross levels of organization (e.g., prototype or domain recruitments) complicating the pyramidal structure and ranked system (pyramid *c*) and affecting the reconstruction of common ancestors. Here, a historical reality challenges the demands of taxonomy and complexity.
(iii) *Complexity:* From a complexity perspective, taxonomy and evolution must align with Bunge’s ‘emergent wholes.’ The evolutionary entities that emerge at each level of the pyramid structure (pyramid *d*) must become cohesive units that integrate the emergent wholes of other levels. In doing so, the emergent wholes become prior states of the evolutionary progression. These inter-level mapping relationships, which can be reticulated, enable both recruitment and modularity and the development of a biomolecular communicative agency (Caetano-Anollés 2023). Under this view, the generation of Lamarckian-like end-directed behaviors arises in different contexts through ‘entanglements’ that foster innovation and independent origins. This challenges the pyramid structure by splitting the multi-level layering structure into separate historical progressions or changing the order of emergences. Here, complexity buildup through innovation challenges taxonomy and evolution.

The ‘problem of ancestors’ is made even more difficult by meta-complication, as taxonomy, evolution, and complexity perspectives impinge on each other to foster new ontological considerations. Bunge’s nine-level conceptualizations can be applied to a same problem, although he suggests his view of levels as grades is the most fruitful (Bunge 1960). Four additional considerations may be important. First, if members of levels are not finite, there are no a priori limits on dividing levels into sublevels. Second, rooting derivative levels into parent levels (e.g., rooting the birth of domains in prototypes) does not mean the process must be conservative. The rise of new properties in the emergent derivative level can be accompanied by loss of properties at the parent level. Third, lower levels can arise from higher levels by percolation of evolutionary constraints. Thus, emergence of novelties can be bidirectional. Finally, the structure of levels need not be static. Their definition is dynamic and changes with time.

## Conclusion

The findings presented in this study offer more than a glimpse into the rich tapestry of cellular evolution. They open new avenues for understanding life’s complexity across levels of organization and geological epochs. The gradual accumulation of loop prototypes, unveiled through a meticulous exploration of evolutionary phases and superkingdoms, carries implications that extend beyond the confines of molecular biology. The delineation of six distinct evolutionary phases, marked by differential accrual of loop prototypes, provides a roadmap for deciphering a vocabulary of protein structure as well as pathways of cellular diversification. From the earliest communal prototypes to the emergence of superkingdoms and modern viruses, this narrative illuminates our understanding of origins of life and evolution.

The loop repertoires of common ancestors reveal their structural and functional complexity but also challenge established notions of ancestral cellular life. Cellular ancestors using RNA molecules as genetic information but lacking a ribosomal biosynthetic machinery and crucial catalytic domains for DNA synthesis needed for transcription demand a transformative shift in our understanding of early genetic processes. The widely popular ancient ‘RNA world’ theory (Fine and Pearlman 2023) must be revisited in light of growing countering evidence. The examination of loop types and their geometric properties unveils a discernible pattern of discovery and reuse of novel building blocks with which to enhance the folding vocabulary of proteins. This has implications for fields beyond molecular evolution, reaching into the realms of materials science and bioengineering, where understanding ancient folding designs may inspire innovative solutions.

We also challenge traditional taxonomic perspectives, proposing a ‛processual’ model that aligns with Woese’s vision. Common ancestors are not mere endpoints but evolving stem lines, representing a dynamic, communal conglomerate that shapes life’s progression. This processual view provides a foundation for interdisciplinary exploration, inspiring new questions and inviting cross-disciplinary action. The impact of our discussions extend into realms where the understanding of ancient biological processes can inform and hopefully redefine our perspectives on the origins and diversity of life.

## Data Availability

All data will be made available upon request.
